# Supplementary material for: Ozymandias: a biodiversity knowledge graph
Source: PeerJ. 2019 Apr 8;7:e6739. doi: 10.7717/peerj.6739 (PMC6459178; doi:10.7717/peerj.6739)
Supplement: Supplemental Information 1 [file peerj-07-6739-s001.docx]

## Supplementary Information

### Publications and identifiers

Get count of number of published works for each year, and number of works with identifiers.

PREFIX xsd: <http://www.w3.org/2001/XMLSchema#>

PREFIX rdf: <http://www.w3.org/1999/02/22-rdf-syntax-ns#>

SELECT ?work_date (COUNT(?w) as ?c) (COUNT(?doi) as ?c_doi) (COUNT(?biostor) as ?c_biostor) (COUNT(?jstor) as ?c_jstor) (COUNT(?pdf) as ?c_pdf)

WHERE

{

?w <http://schema.org/datePublished> ?work_date .

# just articles

?w <http://www.w3.org/1999/02/22-rdf-syntax-ns#type> <http://schema.org/ScholarlyArticle> .

# DOI?

OPTIONAL {

?w <http://schema.org/identifier> ?doi .

?doi <http://schema.org/propertyID> "doi" .

}

# BioStor?

OPTIONAL {

?w <http://schema.org/identifier> ?biostor .

?biostor <http://schema.org/propertyID> "biostor" .

}

# JSTOR?

OPTIONAL {

?w <http://schema.org/identifier> ?jstor .

?jstor <http://schema.org/propertyID> "jstor" .

}

# PDF?

OPTIONAL {

?w <http://schema.org/encoding> ?pdf .

?pdf <http://schema.org/fileFormat> "application/pdf" .

}

FILTER regex(?work_date, "^[0-9]{4}$")

#FILTER (xsd:integer(?work_date) > 1980)

}

GROUP BY ?work_date

ORDER BY ?work_date

Data in publications.tsv

### Journal ranks

Query to retrieve top 10 journals for a given decade (in this case 1910)

PREFIX rdfs: <http://www.w3.org/2000/01/rdf-schema#>

PREFIX tc: <http://rs.tdwg.org/ontology/voc/TaxonConcept#>

SELECT ?journal ?issn (COUNT(?journal) AS ?count) WHERE

{

?work <http://www.w3.org/1999/02/22-rdf-syntax-ns#type> <http://schema.org/ScholarlyArticle> .

?work <http://schema.org/isPartOf> ?container .

?container <http://schema.org/name> ?journal .

?work <http://schema.org/datePublished> ?year .

OPTIONAL {

?container <http://schema.org/issn> ?issn .

}

FILTER ((xsd:integer(?year) >= 1910) && (xsd:integer(?year) < " . ($year + 9) . "))

}

GROUP BY ?journal ?issn

ORDER BY DESC(?count)

LIMIT 10

Repeat this query for all decades, aggregate results, then filter for journals with > 200 articles.

Data in journals.tsv

### Citation patterns

Find all pairs of citing articles and get dates they were published.

PREFIX xsd: <http://www.w3.org/2001/XMLSchema#>

SELECT ?cited_identifier_type (xsd:integer(?w_year) as ?from) (xsd:integer(?work_year) as ?to)

WHERE

{

?w <http://schema.org/identifier> ?identifier .

?w <http://schema.org/name> ?w_name .

?w <http://schema.org/datePublished> ?w_year .

# Identifier (e.g., DOI) for work we are displaying

?identifier <http://schema.org/value> ?identifier_value .

?citing_identifier <http://schema.org/value> ?identifier_value .

?citing <http://schema.org/identifier> ?citing_identifier .

# What does this work cite (typically from CrossRef data)

?citing <http://schema.org/citation> ?cited .

# Translate the citing work\'s DOI (or other identifier) into AFD identifier

# Get identifier (typically a DOI) for citing work

?cited <http://schema.org/identifier> ?cited_identifier .

?cited_identifier <http://schema.org/value> ?cited_identifier_value .

?cited_identifier <http://schema.org/propertyID> ?cited_identifier_type .

# Get work(s) with this identifer (may have > 1 if we have CrossRef record in our triple store

?work_identifier <http://schema.org/value> ?cited_identifier_value .

?work <http://schema.org/identifier> ?work_identifier .

?work <http://schema.org/name> ?name .

?work <http://schema.org/datePublished> ?work_year .

# Just include citing records that are also in ALA

FILTER regex(str(?work),\'biodiversity.org.au\') .

FILTER regex(str(?w),\'biodiversity.org.au\') .

FILTER regex(?w_year, "^[0-9]{4}$")

FILTER regex(?work_year, "^[0-9]{4}$")

}

Data in cites.tsv

### Weevils

Number of accepted taxon names per year.

PREFIX rdfs: <http://www.w3.org/2000/01/rdf-schema#>

SELECT ?year (COUNT(?taxonName) AS ?count)

WHERE

{

VALUES ?root_name {"CURCULIONOIDEA"}

?root <http://schema.org/name> ?root_name .

?child rdfs:subClassOf+ ?root .

?child rdfs:subClassOf ?parent .

?child <http://schema.org/name> ?child_name .

?parent <http://schema.org/name> ?parent_name .

?child <http://taxref.mnhn.fr/lod/property/hasReferenceName> ?taxonName .

?taxonName <http://rs.tdwg.org/ontology/voc/TaxonName#rankString> "species" .

?taxonName <http://rs.tdwg.org/ontology/voc/TaxonName#year> ?year .

}

GROUP BY ?year

ORDER BY ?year

Sum these to generate cumulative total.

Number of weevil names published each year:

PREFIX rdfs: <http://www.w3.org/2000/01/rdf-schema#>

SELECT ?year (COUNT(DISTINCT ?name) AS ?c)

WHERE

{

VALUES ?root_name {"CURCULIONOIDEA"}

?root <http://schema.org/name> ?root_name .

?child rdfs:subClassOf+ ?root .

?child rdfs:subClassOf ?parent .

?child <http://schema.org/name> ?child_name .

?parent <http://schema.org/name> ?parent_name .

?child <http://taxref.mnhn.fr/lod/property/hasReferenceName>|<http://taxref.mnhn.fr/lod/property/hasSynonym> ?taxonName .

?taxonName <http://rs.tdwg.org/ontology/voc/TaxonName#rankString> "species" .

?taxonName <http://schema.org/name> ?name .

?taxonName <http://rs.tdwg.org/ontology/voc/TaxonName#year> ?year .

}

GROUP BY ?year

ORDER BY ?year

Combined data in weevils.tsv

### Snails

Number of accepted taxon names per year

PREFIX rdfs: <http://www.w3.org/2000/01/rdf-schema#>

SELECT ?year (COUNT(?taxonName) AS ?count)

WHERE

{

VALUES ?root_name {"CAMAENIDAE"}

?root <http://schema.org/name> ?root_name .

?child rdfs:subClassOf+ ?root .

?child rdfs:subClassOf ?parent .

?child <http://schema.org/name> ?child_name .

?parent <http://schema.org/name> ?parent_name .

?child <http://taxref.mnhn.fr/lod/property/hasReferenceName> ?taxonName .

?taxonName <http://rs.tdwg.org/ontology/voc/TaxonName#rankString> "species" .

?taxonName <http://rs.tdwg.org/ontology/voc/TaxonName#year> ?year .

}

GROUP BY ?year

ORDER BY ?year

Sum these to generate cumulative total.

Number of snail names published each year:

PREFIX rdfs: <http://www.w3.org/2000/01/rdf-schema#>

SELECT ?year (COUNT(DISTINCT ?name) AS ?c)

WHERE

{

VALUES ?root_name {"CAMAENIDAE"}

?root <http://schema.org/name> ?root_name .

?child rdfs:subClassOf+ ?root .

?child rdfs:subClassOf ?parent .

?child <http://schema.org/name> ?child_name .

?parent <http://schema.org/name> ?parent_name .

?child <http://taxref.mnhn.fr/lod/property/hasReferenceName>|<http://taxref.mnhn.fr/lod/property/hasSynonym> ?taxonName .

?taxonName <http://rs.tdwg.org/ontology/voc/TaxonName#rankString> "species" .

?taxonName <http://schema.org/name> ?name .

?taxonName <http://rs.tdwg.org/ontology/voc/TaxonName#year> ?year .

}

GROUP BY ?year

ORDER BY ?year

Combined data in snails.tsv

### Authors and ORCIDs

How many authors of works with DOIs post 2011?

SELECT (COUNT(DISTINCT ?creator) as ?c)

WHERE

{

GRAPH <https://biodiversity.org.au/afd/publication> {

?work <http://schema.org/identifier> ?identifier .

?identifier <http://schema.org/propertyID> "doi" .

?identifier <http://schema.org/value> ?doi .

?work <http://schema.org/datePublished> ?datePublished .

?work <http://schema.org/creator> ?role .

?role <http://schema.org/roleName> ?roleName .

?role <http://schema.org/creator> ?creator .

?creator <http://schema.org/name> ?name .

}

FILTER (xsd:integer(?datePublished) > 2011)

}

How many authors of works with DOIs post 2011 had an ORCID?

SELECT DISTINCT ?orcid_creator

WHERE

{

GRAPH <https://biodiversity.org.au/afd/publication> {

?work <http://schema.org/identifier> ?identifier .

?identifier <http://schema.org/propertyID> "doi" .

?identifier <http://schema.org/value> ?doi .

?work <http://schema.org/datePublished> ?datePublished .

?work <http://schema.org/creator> ?role .

?role <http://schema.org/roleName> ?roleName .

?role <http://schema.org/creator> ?creator .

?creator <http://schema.org/name> ?name .

}

GRAPH <https://orcid.org>

{

?orcid_identifier <http://schema.org/value> ?doi .

?orcid_work <http://schema.org/identifier> ?orcid_identifier .

?orcid_work <http://schema.org/creator> ?orcid_role .

?orcid_role <http://schema.org/roleName> ?orcid_roleName .

?orcid_role <http://schema.org/creator> ?orcid_creator .

?orcid_creator <http://schema.org/name> ?orcid_name .

}

FILTER(?roleName = ?orcid_roleName)

FILTER (xsd:integer(?datePublished) > 2011)

}
